# Adipose Stem Cell-Based Clinical Strategy for Neural Regeneration: A Review of Current Opinion

**DOI:** 10.1155/2019/8502370

**Published:** 2019-11-19

**Authors:** Yu-hao Wang, Yu-chen Guo, Dian-ri Wang, Ji-yuan Liu, Jian Pan

**Affiliations:** ^1^State Key Laboratory of Oral Disease, West China Hospital of Stomatology, Sichuan University, Chengdu 610041, China; ^2^National Clinical Research Center for Oral Diseases & Department of Oral and Maxillofacial Surgery, West China Hospital of Stomatology, Sichuan University, Chengdu 610041, China; ^3^National Engineering Laboratory for Oral Regenerative Medicine, West China Hospital of Stomatology, Sichuan University, Chengdu, Sichuan Province 610041, China

## Abstract

Nerve injury is a critical problem in the clinic. Nerve injury causes serious clinic issues including pain and dysfunctions for patients. The disconnection between damaged neural fibers and muscles will result in muscle atrophy in a few weeks if no treatment is applied. Moreover, scientists have discovered that nerve injury can affect the osteogenic differentiation of skeletal stem cells (SSCs) and the fracture repairing. In plastic surgery, muscle atrophy and bone fracture after nerve injury have plagued clinicians for many years. How to promote neural regeneration is the core issue of research in the recent years. Without obvious effects of traditional neurosurgical treatments, research on stem cells in the past 10 years has provided a new therapeutic strategy for us to address this problem. Adipose stem cells (ASCs) are a kind of mesenchymal stem cells that have differentiation potential in adipose tissue. In the recent years, ASCs have become the focus of regenerative medicine. They play a pivotal role in tissue regeneration engineering. As a type of stem cell, ASCs are becoming popular for neuroregenerative medicine due to their advantages and characteristics. In the various diseases of the nervous system, ASCs are gradually applied to treat the related diseases. This review article focuses on the mechanism and clinical application of ASCs in nerve regeneration as well as the related research on ASCs over the past decades.

## 1. Introduction

Nerve injury is common in the clinic and leads to many other complications, such as muscle atrophy and abnormal bone reconstruction. The treatments of nerve injury cost USA medical insurance $150 billion every year, and these diseases affect 20 million Americans' lives [1]. Nerve injury occurs in 2% to 3% of citizens, and more than 50,000 peripheral nerve injury repair operations are performed per year in the United States [2]. Therefore, nerve injury and its complications cause huge financial burdens for social development and affect patients' life quality. Thus, it is critical for clinicians to solve these urgent problems.

Nerve injury results in muscle atrophy and abnormal bone reconstruction which leads motor dysfunction. In general, satellite cells, as stem cells in skeletal muscle tissue, can repair atrophied and damaged skeletal muscles [3–7]. However, the recovery of damaged musculoskeletal tissue requires the involvement of nerve endings. It will form scar tissues without the involvement of nerve endings [8]. The loss of axonal continuity, nerve demyelination, and neuron cell death after nerve injury can lead to the denervation of skeletal muscle [2]. Some studies have demonstrated that muscle atrophy will happen after denervation within 2 weeks [9]. Furthermore, the accumulation ability of skeletal stem cells (SSCs) will decrease in the mandible with inferior alveolar nerve injury according to the Annual Clinical Congress of the American College of Surgeons in Boston, May 2018 [10]. Scientists attending the meeting have proved that nerve injury can affect the osteogenic differentiation of SSCs and delay the procedure of bone fracture repair [10]. The mandible is the core component of the masticatory system, and any damage to the mandible can cause masticatory muscle disorder. The recovery of damaged nerve may have a positive impact on the bone fracture repair, and briefly, it may provide a new strategy for skeletal muscle dysfunction and bone diseases.

The orthodox treatment for nerve injury can be divided into two major categories: surgical methods and nonsurgical methods. However, both surgical and nonsurgical methods have their own limitations. For example, Robinson et al. found that only 4 of 53 patients who underwent neurological direct suture had some degree of recovery [11]. The possible reason is that the length of nerve defect is so long that the sutured nerve has a large tension between the sutural endings. The majority of clinicians reject to use medication alone for treatment due to the long periodicity of drug therapy. At present, there are no effective methods to treat nerve injury in the clinic. Fortunately, the research on stem cells and tissue engineering in the past decades may make it possible.

## 2. Stem Cells

Stem cells can self-renew and differentiate into multiple lineages. Currently, scientists have isolated several kinds of adult stem cells, such as bone marrow mesenchymal stem cells (BM-MSCs), skeletal stem cells (SSCs), dental pulp stem cells (DPSCs), adipose stem cells (ASCs), neural stem cells (NSCs), fetal-derived stem cells (FDSCs), human periapical cyst-mesenchymal stem cells (hPCy-MSCs), induced pluripotent stem cells (iPSCs), skin epidermal stem cells (SESCs), human amniotic-mesenchymal stem cells (hAMSCs), and hair follicle stem cells (HFSCs) [12–14].

Stem cells in different tissues can expand their quantities by symmetrical division during the growth and development of the human body. Meanwhile, stem cells can self-renew and have great ability of multidirectional differentiation to replace damaged cells by asymmetric division when some injuries occur in different tissues. It has been reported that intravenous injection of MSCs can treat acute lung and kidney injuries in preclinical trials with mouse disease models [15, 16]. ASCs derive from adipose tissues with some shared characteristics of all stem cells. More importantly, it is potential for ASCs to repair damaged tissues including nervous tissues.

## 3. The Fate and Biological Characteristics of ASCs

Easy obtainable methods with little damage for stem cell harvesting are the main ambition. The quantity of ASCs in adipose tissues is 100- to 500-fold compared with that of MSCs in bone marrow tissues. There are two types of human adipose tissue: white adipose tissue and brown adipose tissue. Subcutaneous adipose tissue in white adipose tissue is the main source of ASCs, and this kind of ASCs has a stronger antiapoptotic ability than ASCs located in brown adipose tissue [17]. However, ASCs from brown adipose tissue more easily undergo skeletal myogenic differentiation in the specific microenvironment [18, 19]. The characteristics of ASCs make them popular in the field of regeneration.

### 3.1. The Obtainable Method and Multipotential Differentiation of ASCs

It is widely accepted that ASCs can be harvested from adipose tissues and have great ability of multidirectional differentiation. With 0.075% collagenase type II digestion, ASCs can be harvested from the stromal vascular fraction (SVF) of adipose tissues. The ingredients of SVF include ASCs (15~30%), endothelial cells (10-20%), pericytes (3~5%), and immune cells (25~45%) [20, 21]. Due to the mesodermal origin of ASCs, they can differentiate into adipogenic, osteogenic, and chondrogenic lineages induced by selective medium *in vitro* [22, 23]. Among them, the neural differentiation of ASCs has attracted scientists' attention and created a new cell-based clinical strategy for neurodegenerative diseases.

### 3.2. Neural Differentiation

Safford et al. firstly induced ASCs to differentiate into neuronal phenotype cells which express nestin and neuronal nuclei protein (NeuN) in 2002 [24]. The inducing and differentiation medium they used for neural differentiation of ASCs contained valproic acid, butyl hydroxyanisole, insulin, and hydrocortisone. However, this chemical method is not suitable to induce differentiation *in vivo* due to its disability to construct the corresponding microenvironment in the body for neural regeneration [25]. Moreover, chemical reagents in the medium can cause some extra damage to tissues, which brings pain to patients.

Biological induction methods are more suitable for repairing damaged tissues *in vivo*. ASCs can be induced to differentiate into neural cells if the medium contains some soluble factors secreted from nerve tissues which include cerebellum, hippocampus, and cerebral cortex [26]. ASCs can also secrete some neurotrophic factors in the process of neural differentiation, such as nerve growth factor (NGF), brain-derived neurotrophic factor (BDNF), glial-derived neurotrophic factor (GDNF), ciliary neurotrophic factor (CNTF), and fibroblast growth factor (FGF) [27]. The current problem lies in how to control the direction of neural differentiation. Both neurons and glial cells can promote the remyelination of nerve which is of great importance for neural regeneration and express the similar markers [28–30]. However, some differences can be pointed out. A higher tendency of neuronal phenotype can be recognized when ASCs are induced by olfactory ensheathing cell conditioned medium (OEC-CM) [31, 32]. Instead, ASCs are more likely to differentiate into glial cells in Schwann cell conditioned medium (SC-CM) with high concentration of GFAP [31, 32].

ASCs can promote axonal regeneration, myelination, and functional recovery [33]. ASCs upregulate the expression of myelin protein zero, peripheral myelin protein-22, and myelin basic protein, which promotes self-regulation of ASC differentiation and reestablishes the connection between damaged nerve and target organs [34]. Some animal models have proved that ASCs have neuroprotection abilities and provide trophic supports for axon regeneration in optic nerve transection, glaucoma, and retinitis pigmentosa [35–37]. Furthermore, ASCs were applied to the preclinical trials for the CNS diseases, such as Alzheimer's disease (AD) and Parkinson's disease (PD) [38].

## 4. The Regulation and Mechanism in the Neural Differentiation of ASCs

There is a complex regulatory network in the neural differentiation of ASCs. In addition to neurotrophic factors secreted by ASCs, parts of signaling pathways are also involved in the differentiation courses. Most importantly, ASCs can promote their own neural differentiation by regulating the microenvironment. It is widely accepted that the microenvironment regulation interacts with the neural differentiation of ASCs [39]. The neural differentiation of ASCs takes place under complex regulatory mechanisms and promotes neural regeneration in order to repair damaged nervous tissues.

### 4.1. The Neurotrophic Effect

The paracrine function of stem cells is a kind of mechanism that accelerates the process of neural differentiation. Undifferentiated ASCs can secrete neuroprotective factors to enhance neural regeneration and reduce muscle atrophy [40, 41]. These neuroprotective factors include BDNF, GDNF, CNTF, and neurotrophin-4 [41]. Furthermore, some angiogenesis and antiapoptotic factors are also secreted by ASCs, including hepatocyte growth factor (HGF), transforming growth factor-*β* (TGF-*β*), and vascular endothelial growth factor (VEGF) [42–46]. Some scientists believe that the inhibition of apoptosis is a crucial step in tissue regeneration, which is decided by the concentration of antiapoptotic factors in the microenvironment [47]. Antiapoptotic factors and neurotrophic factors can promote neuron proliferation and survival [48, 49]. Another important point is that the activation and proliferation of microglia accelerate the process of some neurodegenerative diseases, for example, traumatic brain injury (TBI) [50–54]. Jha et al. have proved that trophic factors secreted by ASCs can normalize microglia and slow down the development of neurodegenerative diseases [55].

Neurotrophic factor is a sort of protein molecule necessary for the growth and survival of neurons [56]. Among the known neurotrophic factors, BDNF is one of the most important factors in the development of the nervous system [41]. BDNF is a neural-related protein encoded early during the embryonic period and its genome is made of eight promoters, each of which binds to a common BDNF full-length protein-encoding exon [57]. Transcription of BDNF is regulated by several mechanisms [58–60]. For example, the cAMP response element-binding protein (CREB) controls the transcription of exon IV and it is a key factor for the synaptic plasticity of neurons and cognition [61–64]. Furthermore, forskolin is a cAMP-elevating agent that can upregulate the expression of BDNF [65]. Recent studies have shown that the use of BDNF has a positive impact on synaptic plasticity, neuron-glia communication, and regulation of neurite outgrowth [66, 67]. The neuron-glia communication is the foundation of neural maintenance. Glial cells can upregulate the expression of BDNF by receiving the signals from neurons [67–69].

### 4.2. The Microenvironment Regulation of ASCs

ASCs can improve the microenvironment for neural regeneration by inhibiting inflammatory responses [70]. Inflammation is induced by proinflammatory immune cells and cytokines and ASCs can secrete angiogenic factors to inhibit that process [71–74]. The newborn blood vessels ameliorate the microenvironment for tissue recovery while the inhibition of angiogenesis aggravates the progression and pathology of inflammation [75]. Black et al. found that ASCs reduced inflammatory response and demonstrated positive therapeutic effects on chronic inflammatory bowel disease in dogs [76]. ASCs can inhibit neural cell apoptosis by releasing anti-inflammatory factors and cytokines, which provide a stable microenvironment for the neural differentiation of ASCs [77].

The main anti-inflammatory factors secreted by ASCs include tumour necrosis factor-inducible gene 6 protein (TSG-6) and STC-1 [78–81]. TSG-6 is a component of the negative feedback mechanism and it can downregulate the inflammatory response [82]. Increasing evidences indicate that some paracrine factors secreted by ASCs are enough to alleviate inflammatory diseases in animal neural disease models [78, 80, 83, 84]. The anti-inflammatory and immunomodulatory effects of these cytokines and chemokines are not only affected by the status of ASCs but are also affected by the concentration of TGF-*β*1, tumour necrosis factor-*α* (TNF-*α*), lipopolysaccharide (LPS), and hypoxia in microenvironments [85–88]. Taken together, the interaction between the neural differentiation of ASCs and the regulation of the microenvironment has a positive influence on inflammatory inhibition.

### 4.3. The Regulation of Signaling Pathways

Since the discovery of the signaling pathways was introduced, scientists have discovered that most of cell physiology can be explained by the regulation of signaling pathways. The neural differentiation of ASCs is also regulated by multiple signaling pathways. Here, we reviewed several known signaling pathways involved in the neural differentiation process.

Researchers have discovered that the Wnt signaling pathway is involved in the formation of the brain. The Wnt/LEF/TCF genes work synergistically to participate in the development of the hippocampal gyrus, and Wnt3a knockout can stop the development of hippocampus in mouse embryos [89–91]. In addition, the Wnt signaling pathway is also involved in the initiation of axon formation. Wnt7a can induce the reconstitution of axons and growth cones in mossy nerve fibers, as well as the collection of receptors I [91]. In contrast, Jang et al. have demonstrated that the classical Wnt signaling pathway does not regulate the neural differentiation of ASCs. Instead, they have proved that the noncanonical Wnt signaling pathway activates the neural differentiation of ASCs by regulating the activation/phosphorylation of Wnt5a/JNK signaling pathway [92].

Some other signaling pathways have been found to participate in the neural differentiation process, such as ROCK and BDNF/TrkB signaling pathway. Ren et al. have proved that the ROCK pathway inhibitor, namely, Y-27632, could accelerate the neural differentiation of ASCs in their experiment. After adding Y-27632 into the culture medium, the shape of mouse ASCs switched to neuronal-like cells. Furthermore, the cells lost their neuron-like morphology once Y-27632 was removed from the medium [93]. It indicates that the ROCK signaling pathway inhibits the neural differentiation of ASCs. Some scholars have pointed out that ASCs may be also regulated by the BDNF/TrkB signaling pathway during the neural differentiation [84]. The BDNF/TrkB signaling pathway induces the secretion of BDNF which is a neurotrophic factor which can promote the neural differentiation of ASCs as previously mentioned [85]. However, scientists are not sure how these multiple signaling pathways interplay in the neural differentiation of ASCs. More research is needed in this field.

## 5. The Prospects for Clinical Application of ASCs in Neural Disease

Due to the limited therapeutic effect of clinical methods, treatment for nerve injury cannot keep pace with the life quality of people. Many elderly people suffer from nervous system diseases around the world. For example, the organic chemical pollution in water always causes serious nervous system diseases in developing countries and backward regions. Nerve injury not only leads to neurological disorders but also musculoskeletal system damage. Taking into account the limitations of current therapeutic methods, the application of stem cell-based therapy is extremely urgent.

The occurrence of neurodegenerative diseases involves a variety of pathophysiological mechanisms that determine the progress and severity of the diseases including neuroinflammation, mitochondrial dysfunction, and protein aggregation [94]. There are several common neurodegenerative diseases in the clinic, such as AD, PD, TBI, and spinal cord injury (SCI). In addition to their own ability to differentiate into nerve cells, ASCs can also secrete various neurotrophic factors and immune regulatory mediators. In the recent years, clinical application of ASCs has attracted much attention in the field of regenerative medicine.

### 5.1. AD

AD is a neurological degenerative disease with family heritability [95]. It is characterized by generalized dementia, such as memory impairment, loss of recognition, abnormal motion, and personality and behavioural change. AD may be a heterogeneous group of diseases, which is regulated by a variety of factors, including biological and psychosocial factors. Entanglement of amyloid-*β* plaques and neuronal fibers, neurodegeneration of the limbic system, and neural progressive decline are the main pathological features of AD [96].

Kim et al. have proved that the application of ASCs in the AD mouse experimental models was feasible [97]. In their study, the Morris water maze test (MWM) of mice was significantly improved. They found that A*β* plaque formations were reduced in the cerebral cortex. Amyloid precursor protein (APP) levels were reduced and A*β*-degrading enzyme levels were also upregulated. These phenomena clearly showed that the symptom of AD has been ameliorated. In another experiment, ASCs increased the secretion of anti-inflammatory factors, enhancing the expression of A*β*-degrading enzymes and raising the response levels in cognitive and memory tests [38]. Furthermore, ASCs increased the secretion of interleukin-10 and induced microglia to polarize the activation phenotype as well as express several vascular and neurotrophic factors [38, 97, 98]. In the recent years, Pérez-González et al. found that ASCs could secrete leptin during neural differentiation. Leptin is a kind of protein hormone which promotes the neural regeneration of stem cells *in vitro* and slows down the process of neurodegenerative diseases *in vivo* [99].

### 5.2. PD

PD is a common degenerative disease of the nervous system in the elderly. The main pathological features of PD are progressive dopaminergic neuron loss in the substantia nigra pars compacta. Tremor, muscle rigidity, and decreased motion are the main clinical features of PD. Zhang et al. have found that PD patients always had chest muscle tissue tension which lead to breath function disorder [100]. It shows that the nervous system damage of PD is not the most fatal factor for patients. Dyspnea and its complications caused by PD are the greatest harm to the human body.

In animal experimental models of PD, ASCs upregulated the secretion of soluble growth factors including anti-inflammatory factors and BDNF [101]. BDNF is an important neurotropic factor which can promote differentiation of stem cells and anti-inflammatory can improve the microenvironment as described earlier. The ASC-based regenerative therapy has a huge potential to treat PD and provides us a new strategy to improve neural and musculoskeletal tissue function for PD patients. However, Schwerk et al. found that the function of regenerative dopaminergic neurons induced by ASCs cannot completely replace that of lost dopaminergic neurons [102]. Furthermore, some scientists demonstrated that ASCs cannot improve the survival rate of PD patients after clinical treatment [103]. More research is required to clarify whether ASCs are useful in the treatment of PD.

### 5.3. SCI

SCI is one of the common symptoms in serious traumas caused by car accidents or falls. Serious injury to the limbs and muscle atrophy due to disconnection between muscles and damaged nerves disturb the ordinary life of patients. Thousands of SCI patients impose a huge burden on the development of social economy, and it costs billions of dollars every year [1]. The prevention, treatment, and rehabilitation of SCI have become a major issue in the medical field due to its urgency for patients and society.

Some studies have shown that ASCs can survive and migrate to damaged nerve tissue in animal experimental models [104]. Meanwhile, transplanted ASCs express GFAP and neuronal nuclear antigens in ischaemic encephalopathy [105]. In a previous study, the expression of GFAP, NF160, and Tuj-1 of ASCs was positive after transplanted ASCs were inserted into lentiviral vectors that were GFP-tagged in SCI models [8]. It is suggested that the implantation of ASCs can differentiate into astrocytes and oligodendrocytes as well as neurons. Neurons deriving from differentiation can convey regenerative information from proximal-disrupting-ending neural fibers to the distal side [106].

The neural regeneration in SCI has a positive effect on innervation to muscle after peripheral nerve injury. When the peripheral nerve breaks down, the skeletal muscle which is innervated by the damaged nerve degenerates and muscle atrophy occurs. The neural transdifferentiation process may be the consequence of cytokine secretion, the interactions of ASCs and intercellular signaling pathways of ASCs [107]. Also, ASCs have been shown to secrete a variety of angiogenic and antiapoptotic cytokines, which support tissue regeneration and minimize tissue damage [108].

### 5.4. TBI

TBI is a type of disruption or alteration of brain function caused by external forces. Skull fracture and intracranial hypertension caused by TBI can lead to conscious disturbance, headache, and vomiting which are transient or long-lasting clinical symptoms of patients. External forces that cause TBI include acceleration or deceleration, direct compression, penetration of objects, and explosion damage. In the United States, the top three causes of TBI are fall (28%), motor vehicle accidents (20%), and pedestrian impact (19%) [109]. Masel and Dewitt thought that TBI is a cascade process involving primary and secondary brain injury instead of a simple external force injury process [110]. Primary damage refers to mechanical damage to the brain tissue caused by external forces. Secondary damage is a cellular metabolic event that occurs after an external force injury [111]. Within 24 hours after the brain tissue injury, the blood-brain barrier has been damaged and inflammatory cells enter the brain tissue leading to the occurrence of inflammation.

Tajiri et al. have proved that ASCs and ASC-associated secreted proteins can reduce cortical damage in mouse TBI models [112]. But the experimental mice were killed at an early stage, and the relevant mechanisms have not been proved. However, a possible mechanism is increasingly supported in scholars: inflammatory suppression theory [113]. Regarding these patients suffering TBI, inflammatory cells release kinds of immune-mediated factors. It is often considered as a secondary brain injury [114, 115]. TNF-*α*, as a kind of inflammatory factor, mainly predominates the inflammatory response [116, 117]. Controlling the inflammatory response after injury can be thought as a target for the TBI treatment. Kappy et al. have demonstrated that ASCs and its own secreting proteins downregulate the secretion of inflammatory factors and inhibit the inflammation in TBI [118–120].

Furthermore, *β*-APP is thought to be an important marker of nerve damage [121]. *β*-APP is a complete membrane protein with a high concentration in neuronal synapses. The role of *β*-APP in the brain has not been clarified, but the concentration of *β*-APP has been found increasing in the mouse TBI model [121]. *β*-APP can be used as a marker for diagnosing nerve damage and assessing the severity of TBI [122]. In Kappy's experiment, inserting ASCs into TBI mouse maintains the *β*-APP concentration instead of making the concentration of *β*-APP continuously increasing [118]. It suggests that ASCs play a neuroprotective role in the TBI model.

## 6. Conclusion

Stem cells have great abilities of multidirectional differentiation and are widely found in nearly all organs and tissues except the heart. When human tissues or organs are damaged or diseased, stem cells can differentiate into corresponding progenitor cells and replenish the cell pools to recover normal function of organs. However, due to the particularity of the nervous system, nerves are less able to self-regenerate. The nerve injury is not only limited to the nerve tissues but also often affects the musculoskeletal tissues. Muscle atrophy and scar formation can be effectively prevented by neural regeneration. At present, the clinical therapy for neurodegenerative diseases mainly includes surgical and nonsurgical means, such as neurolysis, direct nerve suture, and drug treatment. Although these methods have been proved to have a degree of curative effect, it has not lived up to expectations. In recent years, stem cell-based therapies are expected to replace orthodox treatment.

ASCs that are isolated from adipose tissue can differentiate into other kinds of cells with a low mortality rate *in vivo* and *in vitro*. ASCs have advantages of easy material extraction, which means that they could be extracted from many types of tissues with slight damage to the body. Most importantly, ASCs are characterized by low immunogenicity and are not susceptible to immune rejection. Considering the advantages above, we are looking forward to a fact that ASCs will play a crucial role in the treatment of various tissue and organ diseases. In particular, in the nervous system, ASCs are important for promoting neural regeneration. The neurogenic and osteogenic differentiation of ASCs accelerates the recovery of damaged tissues. It will provide a new method for orthodox treatments. However, there are still many issues in the field of neural regeneration with ASCs. For example, the neural differentiation ability of ASCs extracted from different tissues should be clarified in order to identify the most efficacious ASC source for neural regeneration. More research is needed in the field for clinical application. We truly believe that ASCs would play a signature role for neural regeneration in the future.

## Abbreviations

AD: Alzheimer's disease
APP: Amyloid precursor protein
ASCs: Adipose stem cells
BDNF: Brain-derived neurotrophic factor
BM-MSCs: Bone marrow mesenchymal stem cells
CNS: Central nervous system
CNTF: Ciliary neurotrophic factor
CREB: cAMP response element-binding protein
DPSCs: Dental pulp stem cells
FDSCs: Fetal-derived stem cells
FGF: Fibroblast growth factor
GDNF: Glial-derived neurotrophic factor
GFAP: Glial fibrillary acidic protein
HGF: Hepatocyte growth factor
HFSCs: Hair follicle stem cells
hPCy-MSCs: Human apical cyst-mesenchymal stem cells
iPSCs: Induced pluripotent stem cells
LPS: Lipopolysaccharide
MSCs: Mesenchymal stem cells
MWM: Morris water maze test
NeuN: Neuronal nuclei protein
NGF: Nerve growth factor
NSCs: Neural stem cells
OEC-CM: Olfactory ensheathing cell conditioned medium
PD: Parkinson's disease
PNS: Peripheral nervous system
SC-CM: Schwann cell conditioned medium
SCI: Spinal cord injury
SESCs: Skin epidermal stem cells
SSCs: Skeletal stem cells
SVF: Stromal vascular fraction
TBI: Traumatic brain injury
TGF-*β*: Transforming growth factor-*β*
TNF-*α*: Tumour necrosis factor-*α*
TSG-6: Tumour necrosis factor-inducible gene 6 protein
VEGF: Vascular endothelial growth factor.
